# Mental disorders among pregnant women during the COVID-19 pandemic. A cross-sectional study

**DOI:** 10.1590/1516-3180.2021.0356.27052021

**Published:** 2021-08-27

**Authors:** Deha Denizhan Keskin, Seda Keskin, Sedat Bostan

**Affiliations:** I MD. Assistant Professor, Department of Obstetrics and Gynecology, Faculty of Medicine, Ordu University, Ordu, Turkey.; II MD. Assistant Professor, Department of Obstetrics and Gynecology, Faculty of Medicine, Ordu University, Ordu, Turkey.; III MD. Professor, Department of Health Management, Faculty of Health Sciences, Ordu University, Ordu, Turkey.

**Keywords:** Mental disorders, Pregnancy, COVID-19, Pandemics, Anxiety, Depression, Sleepiness, Psychiatric disorders, Gestation, Coronavirus disease-19, Hopelessness

## Abstract

**BACKGROUND:**

Pregnancy is the most important event in women’s lives and can lead to psychological lability. Several risk factors (such as disasters, events and pandemics) have been correlated with greater prevalence of mental disorders during pregnancy.

**OBJECTIVES:**

To research how pregnant women have been affected by the coronavirus disease-19 (COVID-19) pandemic process, in order to contribute to the limited literature.

**DESIGN AND SETTING:**

Cross-sectional survey study conducted at the Training and Research Hospital of the Faculty of Medicine, University of Ordu, Ordu, Turkey, from February 1 to March 1, 2021.

**METHODS:**

In total, 356 pregnant women were enrolled and completed the survey. Intention of going to hospital and the Beck anxiety, Beck depression, Beck hopelessness and Epworth sleepiness scales were applied to detect mental disorders.

**RESULTS:**

Among the participants, the anxiety, depression, hopelessness and sleepiness scores were 29.2%, 36.2%, 58.1% and 11.8%, respectively. The pregnant women stated that they avoided going to hospital in unnecessary situations by obeying the ‘stay at home’ calls, but also stated that they were afraid of the potential harmful effects of inadequate physician control. However, most of them stated that they would go to the hospital in emergencies.

**CONCLUSIONS:**

This paper illustrated the effect of the COVID-19 pandemic on the mental health of pregnant women and emphasized their high rates of anxiety, depression, hopelessness and sleepiness. Since presence of mental disorders is indirectly related to poor pregnancy outcomes, preventive strategies should be developed, especially during this pandemic process.

## INTRODUCTION

The coronavirus disease-19 (COVID-19) has spread rapidly throughout the world. After it was declared to be a pandemic by the World Health Organization (WHO) on March 11, 2020, the disease became a threat to global health.^1^ In addition to the life-threatening situation of the virus, its economic, social and psychological effects have also been the subject of various studies.^2^^,^^3^ Although vaccination continues rapidly, there is no indication that the COVID-19 pandemic will end soon, according to health authorities. And in this crisis environment, pregnant women still have to give birth during the pandemic.^4^

Pregnancy and childbearing are important events in women’s lives, and both of these can lead to psychological lability. In addition to the anxiety caused by pregnancy itself, several other risk factors (such as disasters, events and pandemics) have been correlated with presence of high anxiety, depression and hopelessness during pregnancy.^5^ Many pandemic-related factors, such as social/physical isolation, lockdown, physical inactivity and economic uncertainty have been causing mental disorders within society. Additional risks specific to pregnancy have made pregnant women more vulnerable to mental disorders than the general population.^6^

On the other hand, as shown in various studies, prenatal mental disorders are associated with miscarriage, preterm birth, low infant birth weight, gestational diabetes and gestational hypertension.^7^^,^^8^^,^^9^^,^^10^

Although many studies revealing the psychological effects of COVID-19 on society have been published, only a limited number of studies investigating the effects of the pandemic on the pregnant population are available in the literature.

## OBJECTIVE

The aim of this study was to investigate how pregnant women have been affected by the pandemic process, in order to contribute to the limited literature on this subject.

## METHODS

This descriptive cross-sectional survey study was conducted between February 1 and March 1, 2021. Over this period, the numbers of cases of COVID-19 infection were increasing rapidly in the city of Ordu, which is the third largest city by population in the Black Sea region of Turkey. Furthermore, also over the period of the survey, this city and its province (Altınordu) ranked highest in the weekly number of cases per 100,000 population in Turkey.^11^

The study population consisted of pregnant women who were followed up at the Ordu University Training and Research Hospital, in Ordu. Our study received ethical approval from the Ministry of Health of the Republic of Turkey, under the number 2020-05-11T18_34_16, dated May 27, 2020.

In our study, data from 356 pregnant women who completed the survey questionnaire were analyzed. Patients diagnosed with COVID-19 or with existing psychiatric diseases were excluded from the study.

The first part of the survey included ten questions that aimed to collect data about the participant’s demographic characteristics and pregnancy conditions. These ten questions asked about their age, number of pregnancies, pregnancy trimester, work, body mass index, education, marital status, presence of chronic disease, smoking and any close contact with COVID-19-infected patients.

The second part of the survey consisted of the “intention of going to hospital” scale. This scale, consisting of nine questions, was developed by Bostan.^12^ The questions in this part were as follows: Currently, while the coronavirus pandemic continues…? (a) I go to the hospital because I am curious about the condition of the patients; (b) I go to the hospital to visit my relatives or friends; (c) I go to the hospital to have my medicine prescribed; (d) I go to the hospital to have the routine tests that I have in mind; (e) I go to the hospital for the appointment that my physician has given me for a routine check; (f) I go to the hospital if I feel mild discomfort; (g) I go to the hospital if my current disease increases a little more; (h) I go to the hospital if my current illness becomes serious; (i) I go to the hospital if I have some trouble that I think is urgent; and (j) I would never go to the hospital. The participants were asked to answer these questions by selecting one of five options that were scored from zero to four. One of the questions on the intention of going to hospital scale was excluded from the analysis because it did not reach a sufficient factor load.

Lastly, in the third part of the survey, the participants answered questions on the Beck anxiety scale,^13^ Beck depression scale,^14^ Beck hopelessness scale,^15^ and Epworth sleepiness scale.^16^ Details of the questionnaire are presented in Table 3. The Beck anxiety scale was developed in 1988 and consists of 21 items on Likert scales ranging from zero to three, and with raw scores ranging from 0 to 63. The scores are classified as minimal anxiety (0 to 7), mild anxiety (8 to 15), moderate anxiety (16 to 25) and severe anxiety (26 to 63).

The Beck depression scale was first introduced in 1961 and contains questions on 21 symptoms and attitudes. The questions receive ratings from zero to three to reflect the intensity of symptoms or attitudes, and this gives rise to a score that can range from 0 to 63. A total score of less than 9 depicts minimal depression; a score of 10-18, mild depression; a score of 19-29, moderate depression; and a score of 30 or above, severe depression.

The Beck hopelessness scale was defined in 1974 and includes 10 questions. Its total score can range from 0 to 20. Scores from 0 to 3 are considered normal; 4 to 8, mild hopelessness; 9 to 14, moderate hopelessness; and greater than 15, severe hopelessness.

The Epworth sleepiness scale was initially reported in 1991 and is a self-administered questionnaire consisting of 8 questions. Respondents are asked to rate each item on a four-point scale (0 to 3). On this scale, scores of 0-9 are evaluated as mild sleepiness and scores of 10 and above are evaluated as severe sleepiness.

The Beck and Epworth scales are widely used within in healthcare. The validity of these scales has been tested by means of confirmatory factor analysis. The validity of the “intention of going to hospital” scale was tested by means of exploratory factor analysis. In addition, reliability analyses were performed on all the scales. The Kaiser-Meyer-Olkin (KMO) sampling coefficient was found to be greater than 0.80 for each scale, and this was accepted as very good.

The results from the Bartlett sphericity test, which was used to evaluate the appropriateness of the scales for factor analysis, were also found to be significant (P = 0.000). According to these evaluations, the scales were found to be suitable for factor analysis. It was understood that the factor loads of all the scales were generally high and their power to explain the total variance was sufficient.

To analyze the results from the study, the Statistical Package for the Social Sciences (SPSS) software, version 26, was used (IBM Statistics, Armonk, New York, United States). The analyses were carried out using a 95% confidence interval (P = 0.05). Descriptive statistical methods and correlation analyses were used in the study.

## RESULTS

In total, 356 pregnant women in Ordu were enrolled in this study. Their demographic characteristics and pregnancy conditions are demonstrated in Table 1. The mean age of these pregnant women was 30.06 + 6.41 years. Among them, the majority were in the 20-25 age group (26.4%), married (92.7%), in the first trimester (39.3%) and primigravid (39.3%). Among all the participants, 65.7% were housewives or not working, 91.9% had a normal body mass index and 48.6% had completed their elementary education (total of eight years). In addition, 19.7% of the participants had a chronic disease and 6.7% were smokers. Only 4.2% of the pregnant women stated that they had had close contact with people diagnosed with COVID-19.

**Table 1.** Demographic characteristics and pregnancy conditions

	n	%
Age		
18-24	79	22.2
25-29	94	26.4
30-34	80	22.5
35-39	74	20.8
40-44	29	8.1
No. of pregnancies		
1	140	39.3
2	95	26.7
3	72	20.2
4	23	6.5
> 5	26	7.2
Pregnancy trimester		
First	140	39.3
Second	95	26.7
Third	72	20.2
Work		
Housewife/not working	234	65.7
Working	122	34.3
Body mass index (BMI)		
Normal weight (BMI < 30)	327	91.9
Obese (BMI > 30)	29	8.1
Education		
Elementary school (eight years)	173	48.6
High school (four years)	125	35.1
University and above	58	16.3
Marital status		
Married	330	92.7
Single or divorced	26	7.3
Presence of chronic disease		
No	286	80.3
Yes	70	19.7
Smoking		
No	332	93.3
Yes	24	6.7
Any close contact with COVID-19 infected patients		
No	341	95.8
Yes	15	4.2

The second part of the survey consisted of eight questions on the “intention of going to hospital” scale, which are presented in Table 2. This scale was designed using a five-degree Likert scale. For as long as the pandemic continues, the participants declared that they would not go to the hospital to visit their relatives or friends, or to have their medicine prescribed, or to have the routine tests that they had in mind (96.6%, 50.8% and 57.3% respectively). The proportion of the patients who stated that they would not go to the hospital even when they felt mild discomfort was 60.7%. More than half of the participants (55.9%) declared that they would go to the hospital for routine checks, provided that an appointment had been made by their physician. About two thirds of the participants (68.8%) said that they would go to the hospital if their illness became serious, while four out of five (80.3%) said that they would go to the hospital if they thought their illness was urgent. 46.1% of the pregnant women showed uncertainty regarding their intention to go to the hospital if their current disease increased a little more.

**Table 2.** “Intention of going to hospital” scale

Question	I will definitely not go		I will not go		I may go		I will go		I will definitely go		$\overset{-}{x}$	SD
	n	%	n	%	n	%	n	%	n	%		
a^*^	factor load was insufficient											
b^*^	198	55.6	146	41.0	6	1.7	6	1.7	0	0.0	0.49	0.62
c^*^	73	20.5	108	30.3	110	30.9	61	17.1	4	1.1	1.48	1.03
d^*^	80	22.5	124	34.8	94	26.4	53	14.9	5	1.4	1.37	1.03
e^*^	17	4.8	30	8.4	110	30.9	156	43.8	43	12.1	2.50	0.97
f^*^	54	15.2	162	45.5	87	24.4	47	13.2	6	1.7	1.40	0.95
g^*^	7	2.0	34	9.6	164	46.1	130	36.5	21	5.9	2.34	0.80
h^*^	0	0.0	12	3.4	99	27.8	182	51.1	63	17.7	2.83	0.75
i^*^	0	0.0	5	1.4	65	18.3	204	57.3	82	23.0	3.01	0.68
j^*^	factor load was insufficient											
factor means											1.76	0.48

$\overset{-}{x}$ = sample mean; SD = standard deviation.

^*^The questions of this scale were as follows: Currently, while the coronavirus pandemic continues…? (a) I go to the hospital because I am curious about the condition of the patients; (b) I go to the hospital to visit my relatives or friends; (c) I go to the hospital to have my medicine prescribed; (d) I go to the hospital to have the routine tests that I have in mind; (e) I go to the hospital for the appointment that my physician has given me for a routine check; (f) I go to the hospital if I feel mild discomfort; (g) I go to the hospital if my current disease increases a little more; (h) I go to the hospital if my current illness becomes serious; (i) I go to the hospital if I have some trouble that I think is urgent; and (j) I would never go to the hospital.

The responses to the Beck anxiety, Beck depression, Beck hopelessness and Epworth sleepiness scales are presented in Table 3. Approximately one in three of the participants (29.2%) stated that they experienced anxiety. The rates of mild, moderate and severe anxiety were 18.3%, 8.1% and 2.8%, respectively. The depression rates were observed to be close to anxiety rates (36.2%). The rates of mild, moderate and severe depression were 25.5%, 7.6% and 3.1%, respectively. The hopelessness rates were higher than the anxiety and depression rates. While 44.3% of pregnant women reported having mild hopelessness, 11.8% experienced moderate and 2% experienced severe hopelessness. Lastly, mild sleepiness was found in 88.2% and severe sleepiness in 11.8% of the pregnant women.

**Table 3.** Beck anxiety, Beck depression, Beck hopelessness and Epworth sleepiness scales

	n	%
Beck anxiety scale		
➢ Minimal anxiety (0-7)	252	70.8
➢ Mild anxiety (8-15)	65	18.3
➢ Moderate anxiety (16-25)	29	8.1
➢ Severe anxiety (26-63)	10	2.8
Beck depression scale		
➢ Minimal depression (0-9)	227	63.8
➢ Mild depression (10-18)	91	25.5
➢ Moderate depression (19-29)	27	7.6
➢ Severe depression (30-63)	11	3.1
Beck hopelessness scale		
➢ Normal (0-3)	149	41.9
➢ Mild hopelessness (4-8)	158	44.3
➢ Moderate hopelessness (9-14)	42	11.8
➢ Severe hopelessness (15-20)	7	2.0
Epworth sleepiness scale		
➢ Mild sleepiness (0-9)	314	88.2
➢ Severe sleepiness (10-24)	42	11.8

Correlation analysis was done to understand the relationships between the research scales is presented in Table 4. It was observed that there was no statistically significant relationship between the intention of going to the hospital and the levels of anxiety, depression, hopelessness and sleepiness. On the other hand, a very strong linear relationship was found between the anxiety, depression, hopelessness and sleepiness levels of the pregnant women at the P = 0.001 error level.

**Table 4.** Correlation analysis between the research scales

	Intention of going to hospital	Beck anxiety	Beck depression	Beck hopelessness
Intention of going to hospital	1			
Beck anxiety	-0.029	1		
Beck depression	-0.041	0.980^**^	1	
Beck hopelessness	-0.039	0.962^**^	0.990^**^	1
Epworth sleepiness	-0.038	0.970^**^	0.993^**^	0.992^**^

^**^Correlation is significant at the 0.01 level (two-tailed).

## DISCUSSION

More than one year has passed since COVID-19 was declared a pandemic. Despite this length of time, the effects of the pandemic are still felt all over the world.^17^ Undoubtedly, healthcare professionals constitute the group most affected in this process. While the effect of pandemic on the healthcare sector and on healthcare workers has been the subject of many studies, the number of investigations in the literature, on its effects on the pregnant population, is limited.^18^^,^^19^^,^^20^ The aim of this descriptive study was to contribute to this limited literature through investigating the effects of the pandemic process on pregnant women.

Pregnancy itself is already known to be a cause of psychological lability, and this has been shown in many studies.^5^^,^^21^^,^^22^^,^^23^ Not surprisingly, pregnant women have relatively high prevalence rates of anxiety, depression, hopelessness and sleepiness. In three different meta-analyses that compiled data on the mental status of pregnant women, the prevalences of anxiety, depression and sleepiness were given as 15.2%, 11.9% and 45.7%, respectively.^24^^,^^25^^,^^26^ The results from our study revealed that 29.2%, 36.2%, 58.1% and 11.8% of the participants had anxiety, depression, hopelessness and sleepiness, respectively, with levels varying from mild to severe. Thus, the pregnant women participating in our study reported that they experienced more anxiety, depression, hopelessness and sleepiness than in reports in the literature. We attributed this excess rate to the high prevalence of ­COVID-19 infection in Ordu province.

In addition, many studies have shown that events such as natural disasters, economic crisis and pandemics adversely affect the mental health of the community.^3^^,^^27^^,^^28^ It is inevitable that such events will affect pregnant women worse than the general population.^22^ It has been determined that the main factors adversely affecting pregnant women’s mental health and causing anxiety in pandemics have been abstention from the daily routine, the need for social distancing and decreased social support. They also expressed fears about their babies’, their families’ and their own health, due to the uncertainties of the pandemic.^6^ Studies on pregnancy in relation to the COVID-19 pandemic have shown that the main factors that increased anxiety among pregnant women were lockdown measures and disruption to routine pregnancy follow-up.^6^^,^^24^^,^^25^ Studies have shown that, in this pandemic situation, pregnant women prefer to have fewer checkups from doctors for fear of infection, but also worry that their pregnancy could be made more complicated through inadequate follow-up.^4^^,^^6^^,^^22^^,^^29^

Our findings showed that 29.2% of the pregnant women experienced anxiety. According to the “intention of going to hospital” scale, the pregnant women participating in this survey stated that they refrained from going to hospital in unnecessary situations such as visiting relatives/friends, having medications prescribed and having the routine tests that they had in mind. This result showed that the pregnant population had reached the required level of awareness regarding COVID-19 by following appeals to ‘stay at home’. But at the same time, their preference to go to routine checks if they got an appointment showed their fear of the potential harmful effects of inadequate physician control. While their hesitation regarding going to hospital continued even if they felt mild discomfort, most of the pregnant women stated that they would go to the hospital in cases of emergency (such as if their current disease worsened or if they thought that their situation was urgent).

We observed that anxiety and depression rates have been found to be related to each other in many studies.^6^^,^^20^^,^^23^ In our study, 36.2% of the participants presented depression (in 10.7%, it was moderate or severe). As expected, anxiety related to COVID-19 infection also led to depressive symptoms in the pregnant population.

In a cross-sectional study investigating sleep quality among pregnant women, sufficient sleep duration was defined as sleeping 7-9 h/day.^30^ In some studies, it has been shown that the proportion of pregnant women with poor sleep quality (for a variety of reasons) was between 38.8% and 82.6%.^31^^,^^32^^,^^33^ In our study, the rate of sleepiness was found to be lower than in the literature (11.8%). We attributed this low rate to the Epworth sleep scale that we used in the survey, because this scale evaluated sleepiness in only two stages, unlike the ones used in the literature. In studies on pregnant women, high anxiety level, low social support and insufficient partner support were found to be associated with low sleep quality. Therefore, it is very important to provide the necessary social and partner support in order to protect pregnant women from the psychologically destructive effects of the pandemic. With this support, situations such as social isolation and postpartum depression can be prevented, and a healthy partner relationship and adequate baby care can be provided after delivery.^34^^,^^35^

Studies conducting subgroup analysis have shown that multigravid pregnant women were more affected by the COVID-19 pandemic. Multigravid women have to struggle with several challenges, such as increased financial responsibilities and having an additional child. Moreover, in these studies, it was observed that greater anxiety was detected in the first and third trimesters, possibly due to hormonal changes and fear of delivery.^36^^,^^37^

Many studies have suggested that mental disorders have a more severe course in the prenatal and postnatal periods and that they have lasting negative effects on mothers, fetuses and children. The adverse prenatal outcomes include preeclampsia, gestational hypertension, gestational diabetes, miscarriage, intrauterine growth restriction and low birth weight.^7^^,^^8^^,^^9^^,^^10^ Emotional, behavioral and cognitive problems have been found to be more common among postnatal mothers. Additionally, in further follow-up, changes to brain structures and functions were observed in their children.^38^^-39^

Some limitations of our study should be noted. Although we found high rates of anxiety, depression, hopelessness and sleepiness among pregnant women in the COVID-19 pandemic, we could not make any comparison with previous times since we did not have pre-pandemic data. In addition, we did not question the main reasons for increases in these parameters during the pandemic period in our survey. Our study provides information about the general population of pregnant woman because we were unable to clearly distinguish any group at higher risk among these pregnant women, through subgroup analyses, due to the limited number of patients and the cross-sectional structure of this survey.

## CONCLUSION

This study revealed the effect of the COVID-19 pandemic on the mental health of pregnant women and highlighted the high prevalence rates of anxiety, depression, hopelessness and sleepiness among this population. Since the increased prevalence of mental disorders consequent to the pandemic is indirectly associated with poor pregnancy outcomes, the necessary support needs to be provided for this group of patients. This study has helped to emphasize the necessity of interventions that can be taken, especially during the pandemic process. Further studies could broaden the knowledge of the mental effects of COVID-19 among pregnant women.
